# Epistasis between the MHC and the RCAα block in primary Sjögren syndrome

**DOI:** 10.1136/ard.2007.075044

**Published:** 2007-09-18

**Authors:** S Lester, C McLure, J Williamson, P Bardy, M Rischmueller, R L Dawkins

**Affiliations:** 1 C Y O’Connor ERADE Village, Canning Vale, Western Australia, Australia; 2 Arthritis Research Laboratory, Hanson Institute, The Royal Adelaide Hospital, Adelaide, South Australia, Australia; 3 Rheumatology Department, The Queen Elizabeth Hospital, Woodville, South Australia, Australia; 4 Transplant Services, Australian Red Cross Blood Service, Adelaide, South Australia

## Abstract

**Objective:**

The RCAα block (Regulators of Complement Activation, 1q32) contains critical complement regulatory genes such as CR1 and MCP. This study examined RCAα block haplotype associations with both disease susceptibility and diversification of the anti-Ro/La autoantibody response in primary Sjögren syndrome (pSS).

**Methods:**

115 patients with pSS and 98 controls were included in the study. 93 of 109 (85%) of the patients with pSS were seropositive for Ro/La autoantibodies. The Genomic Matching Technique (GMT) was used to define RCAα block ancestral haplotypes (AH).

**Results:**

RCAα block haplotypes, AH1 and AH3, were both associated with autoantibody-positive pSS (p = 0.0003). Autoantibody associations with both HLA DR3 and DR15 have been previously defined. There was an epistatic interaction (p = 0.023) between RCAα AH1 and HLA DR3, and this genotypic combination was present in 48% of autoantibody-positive patients with pSS compared with 8% of controls. This epistasis is most simply attributable to an interaction between C4 and its receptor, CR1, encoded within the RCAα block. Both DR3 and a relative C4 deficiency are carried on the major histocompatibility complex 8.1 ancestral haplotype. Only four of 92 (4%) autoantibody-positive patients with pSS did not carry any risk RCAα or HLA haplotype, compared with 36 of 96 (38%) controls, and there were differences in haplotype frequencies within autoantibody subsets of pSS.

**Conclusions:**

Normal population variation in the RCAα block, in addition to the major histocompatibility complex, contributes genetic susceptibility to systemic autoimmune disease and the autoantibody response. This finding provides evidence for the role of regulation of complement activation in disease pathogenesis.

Primary Sjögren syndrome (pSS) is a systemic autoimmune disease, which shares a number of clinical, serological and genetic features with systemic lupus erythematosus (SLE).^1^ One of the features of this disease is the high prevalence of autoantibodies to the Ro and La components of a ribonuclear protein (RNP) complex. Ro/La autoantibodies occur in individual patients as targeting either Ro alone, or with specificity to both Ro and La, and autoantibody subgroups are stable over time. Major histocompatibility complex (MHC) disease associations have been known for many years,^2^ ^3^ and more recently it has been recognised that these MHC haplotypes regulate the diversification of the autoantibody response.^4^ ^5^ Ro/La autoantibodies occur in other rheumatological diseases such as SLE; however, they are highly specific for pSS and constitute one of the classification criteria for this disease.^6^ Serum Ro/La autoantibodies have been shown to pre-date disease onset, often for many years, and seropositivity predicts disease severity and extraglandular features in patients with pSS.^7^ More recently, a pathogenic role has been proposed as Ro/La autoantibodies form nucleic-acid-containing immune complexes that can trigger prolonged type I interferon production, leading to a self-perpetuating autoimmune reaction.^8^

Deficiencies in the classical pathway of the complement system have been implicated in the aetiology and pathogenesis of autoimmune diseases such as SLE.^9^ Human complement receptor 1 (CR1, CD35) is an integral membrane complement control protein (CCP) whose primary role on erythrocytes is the non-inflammatory clearance of immune complexes opsonised with C3a and C4b. There is longstanding evidence, over 25 years in our own papers,^10^ for an acquired CR1 deficiency in SLE. A recent meta-analysis has demonstrated an association between a molecular weight variant of CR1 and SLE.^11^ Further, in the mouse, CR1/CR2 (encoded by alternatively spliced forms of the same gene) is a lupus susceptibility gene^12^ ^13^ important in the modulation of the antinuclear autoantibody response.^14^ ^15^

Human CR1 is located at 1q32 in the regulators of complement activation (RCA) complex, which also includes other complement regulatory encoding genes, namely factor H (CFH), complement 4 α and β binding protein’s (C4ABP and C4BBP), decay accelerating factor (DAF, CD56), complement receptor 2 (CR2, CD21) and membrane cofactor protein (MCP, CD46).

Encompassing some 13 Mb of the long arm of chromosome 1, the RCA does not contain an even distribution of CCPs. In fact all CCPs can be found within one of two distinct blocks, located at the telomeric and centromeric ends of the cluster, we refer to these as the α and β blocks respectively. Both blocks are about 500 kb in length and are characterised by extensive imperfect genomic duplication and degeneracy generated by insertions and deletions (indels) such as those created through domain duplication/deletion and retroviral insertion. CR1 is encoded within the RCAα block, where it has been duplicated as part of a segment along with MCP-Like, to form CR1-L and MCP (or vice versa). Segment A (CR1 and MCP-L) and B (CR1-L and MCP) are located next to each other and encompass ∼350 kb of the α block.^16^

As demonstrated some time ago within the MHC,^17^^–^19 and more recently throughout the entire genome,^20^^–^22 genomic blocks are normally defined by gene and segmental duplications while also exhibiting other characteristics such as suppression of recombination and an increased frequency of copy number, indel and single nucleotide polymorphisms.^23^ A consequence of recombination suppression is that haplotypes of these regions are likely to have been inherited faithfully over many generations (ie, ancestral haplotypes (AH)) and will therefore be important in defining complex genetic interactions.^24^

We recently reported a novel haplotyping approach, capable of interrogating the genetically complex RCAα block, by utilising the genomic duplication of CR1 and MCP (segment A and segment B).^25^ The assay uses the principles of the Genomic Matching Technique,^23^ used by us, and other groups, to interrogate the highly complex MHC region for the identification of donor/recipient matches prior to bone marrow transplantation.^26^^–^29 More recently the technique has been extended to forensic applications^30^ and used within the RCA complex on chromosome 1^25^ and the class II region of the canine MHC.^31^ The technique involves the specific amplification, with a single primer pair, of multiple complex geometric elements, all linked and located within duplicated segments. The conceptual basis behind the technique is that duplicated elements make excellent haplospecific markers as they have evolved in line with the many other changes that have also occurred on each haplotype.

The aim of this study was to evaluate RCAα block haplotypes, as defined by the Genomic Matching Technique (GMT), in relation to both disease susceptibility and Ro/La autoantibody responses in patients with pSS. Specifically we hypothesised that genetic variation in the RCAα, in combination with HLA, would influence the diversification of the Ro/La autoantibody response.

## MATERIALS AND METHODS

### Study participants

Ninety-eight Caucasian controls and 115 Caucasian patients with pSS from the South Australian Sjögren syndrome research registry were included in the study. All patients met the revised 2002 American-European consensus research classification criteria for pSS,^6^ and controls were recruited from the same population base as the patients. Anti-Ro/La autoantibody specificity was determined by enzyme-linked immunosorbent assay (RELISA ANA Screening System, Immuno Concepts NA, Sacramento, California, USA) using recombinant Ro60 and La proteins, as part of standard diagnostic procedure.  Sera from patients with anti-La were further tested by counterimmunoelectrophoresis (CIEP)^32^ to confirm whether or not anti-La antibodies detected by enzyme-linked immunosorbent assay were able to be detected by this method.  HLA typing of patients with pSS (serological HLA-B and molecular DRB1) was performed by the Transplantation Laboratory, Australian Red Cross Blood Service, SA Division. Molecular DRB1 typing of the controls was performed by Conexio Genomics (Applecross, WA, Australia). The study was approved by the Human Ethics Committee of The Queen Elizabeth and Royal Adelaide Hospitals and all patients gave informed, written consent.

### RCAα block haplotyping

Haplotypes of the RCAα block were obtained using the GMT assays as previously described.^25^ Briefly, two separate polymerase chain reaction reactions using primer sets CR1MCP5&6 and CR1MCP11&12 were performed on each genomic DNA sample. Each primer set was designed to amplify a duplicated element, located within the CR1 region of both segments (segment A containing CR1 and MCP-Like and segment B containing CR1-Like and MCP). The resulting mix of polymerase chain reaction products were used to define the haplotypic variation within the RCAα block. The polymerase chain reaction products were separated on the basis of size on a Corbett Research GS-3000 automated gel analysis system (Corbett Research (Australia), Mortlake, NSW, Australia). Haplotype assignment and nomenclature are as previously described.^25^

### Statistical analysis

Contingency table analysis of haplotype and genotype frequencies in patients with pSS versus controls was performed by multivariate logistic regression using both additive and dominant allele coding. Associations were further reported as odds ratios (OR) with 95% confidence intervals (CI) obtained by back transformation of the regression coefficients.

## RESULTS

### RCAα block haplotype diversity

We have previously demonstrated substantial polymorphism in GMT RCAα block haplotypes.^25^ More than 20 haplotypes have been defined, although the majority are rare. In the current study of 213 Caucasians (pSS and controls combined), there were three relatively common haplotypes (AH1, AH2 and AH3 as designated by McLure *et al*^25^ each with a frequency of >10%. These three haplotypes combined accounted for 56% of the total haplotypes in the sample. There were a further 14 haplotypes with a frequency between 1 and 3%. These frequencies were considered too low to be informative given the study sample sizes and were therefore combined for analysis purposes. RCAα block genotypes were in Hardy–Weinberg equilibrium in both patients with pSS and controls (p = 0.93, p = 0.21 respectively, exact test).

### RCAα block haplotypes are associated with Ro/La autoantibody-positive primary Sjögren syndrome

CR1 haplotype frequencies were significantly different between patients with pSS and controls (global χ^2^ = 14.6, df = 3, p = 0.002, table 1). Both AH1 (OR 2.1, 95% CI 1.3, 3.3) and AH3 (OR 2.4, 95% CI 1.3, 4.4) were significantly increased in pSS relative to controls implying an association between both of these haplotypes and susceptibility to pSS. However, this association was primarily attributable to the Ro/La autoantibody-positive subgroup of patients with pSS (n = 96, p = 0.0003) and was not evident in the relative minority of patients with pSS who were autoantibody negative (n = 19, p = 0.68).

**Table 1:** RCAα haplotype frequencies in patients with pSS compared with controls

Haplotype	pSS(2N = 230)	Controls(2N = 196)	OR (95% CI)	p Value
AH1	80 (34.8%)	46 (23.5%)	2.1 (1.3,3.3)*	0.002
AH2	25 (10.9%)	27 (13.8%)	1.3 (0.6,2.1)	0.75
AH3	38 (16.5%)	19 (9.7%)	2.4 (1.3,4.4)	0.006
Other	87 (37.8%)	104 (53.1%)	1	
	Global test: χ^2^ = 14.6, df = 3, p = 0.002			

There were three common RCAα block ancestral haplotypes, AH1, AH2 and AH3. “Other” haplotypes, with a frequency between 1 and 3%, were combined for analysis purposes. Both AH1 and AH3 frequencies were increased in patients with pSS relative to controls. This association was attributable to the Ro/La autoantibody-positive subgroup of patients with pSS and was not evident in the minority of patients with pSS who were autoantibody negative (2N = 38).

**Figure 1:**
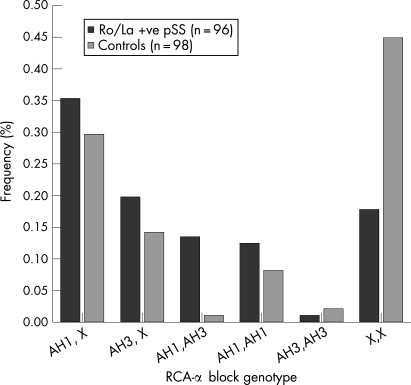
RCAα ancestral haplotype (AH) 1 and AH3 genotype frequencies in patients with primary Sjögren syndrome who were Ro/La autoantibody positive compared with controls. There was a significant departure from an additive or allele dose model (p = 0.043) by logistic regression analysis, and the data are most consistent with AH1 and AH3 exerting dominant effects of similar size. Under the dominant model, AH1 and AH3 appear to be independently associated with autoantibody-positive primary Sjögren syndrome, ie, with multiplicative risks.

With the exception of the relatively rare AH3 homozygotes, the frequencies of all genotypes carrying either AH1 or AH3 were increased in Ro/La autoantibody-positive patients with pSS compared with controls (fig 1). This was accompanied by a striking concomitant decrease in the frequency of all other (X,X) genotypes. There was significant departure from an additive or allele dose model (p = 0.043) by logistic regression analysis, and the data were most consistent with both AH1 and AH3 exerting dominant genotypic effects. Under the dominant model, AH1 and AH3 appeared to be independently associated with autoantibody-positive pSS as there was no evidence of any interaction (p = 0.258). The odds ratios for the dominant genotypic association with autoantibody-positive pSS were 3.8 (95% CI 2.0, 7.3, p = 0.0001) for AH1 and 4.1 (95% CI 1.9, 8.7, p = 0.0003) for AH3. The association for the compound heterozygous AH1,AH3 genotype was predicted by multiplication of these odds ratios.

### Epistatic interaction between the RCAα block and the major histocompatibility complex in susceptibility to Ro/La autoantibody-positive pSS

An association between both HLA-DRB1*0301 (DR3) and HLA-DRB1*1501 (DR15) and autoantibody-positive pSS is well established in Caucasians.^4^ ^5^ Therefore it was of interest to examine relationships between MHC and RCAα block associations with autoantibody-positive pSS.

**Figure 2:**
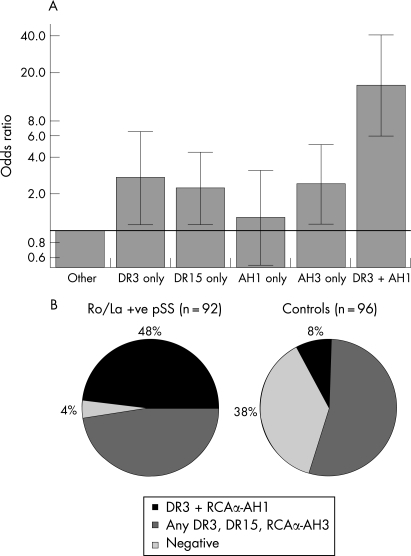
Epistatic interaction between the MHC and RCAα block in Ro/La autoantibody-positive patients with primary Sjögren syndrome (pSS).  (A) Odds ratios (*y*-axis, logarithmic scale) derived by logistic regression for the cross-classification of HLA DR3, DR15 and RCAα AH1, AH3 genotypic combinations (dominant coding) in Ro/La autoantibody-positive patients with pSS (n = 92) relative to controls (n = 98). The vertical bars represent 95% confidence intervals, and the horizontal line represents an odds ratio of 1 (no effect). HLA DR3, DR15 alleles and the RCAα AH3 haplotype were independent risk factors for autoantibody-positive pSS (ie, multiplicative risks), but there was an epistatic interaction between HLA DR3 and RCAα AH1 (interaction term p = 0.021). The genotypic combination of HLA DR3 and RCAα AH1 was the greatest genetic risk factor for autoantibody-positive pSS (OR 15.7, p = 10−8), but in the absence of DR3, there was no effect of RCAα AH1. (B) Pie chart depicting the relative proportions of risk genotypes. The HLA DR3–RCAα AH1 epistatic combination was present in 48% of autoantibody-positive patients with pSS compared with 8% of controls. The majority of other patients with pSS carried any combination of HLA DR3, DR15 and RCAα AH3.

Analysis of the cross-classification of HLA-DR3, DR15 and RCAα AH1 and AH3 genotypic combinations in autoantibody-positive patients with pSS (n = 92) versus controls (n = 96) was performed by multivariate logistic regression using dominant coding and all two-factor interaction terms were evaluated. The odds ratios from this analysis are illustrated in fig 2A. HLA DR3, DR15 alleles and the RCAα AH3 haplotype were independent risk factors for autoantibody-positive pSS, with similar effect sizes. Therefore, the association for compound heterozygous genotypes was predicted by multiplication of these odds ratios. However, there was a disproportionately increased frequency of patients with pSS who carried both HLA DR3 and RCAα AH1 (interaction term, p = 0.021) demonstrating an epistatic relationship.^33^ In the absence of RCAα AH1, HLA DR3 was still associated with Ro/La-positive pSS (OR 2.7, p = 0.025), although the association was greatly enhanced by the presence of AH1 in the same individual (OR 15.7, p = 10^−8^). Conversely, the RCAα AH1 association was not associated with genetic susceptibility to pSS in the absence of DR3 (OR = 1.3, p = 0.6). Moreover, this epistatic genotypic combination of HLA DR3 and RCAα AH1 was the largest genetic risk factor for autoantibody-positive pSS. It was highly specific for autoantibody-positive pSS as it was present in 44 of 92 (48%) patients with pSS who were Ro/La autoantibody positive compared with only eight of 96 (8%) controls (fig 2B). The majority of patients with pSS who did not carry this epistatic genetic risk factor carried any combination of DR3/DR15/AH3, the other risk genotypes identified in the analysis. Therefore, only four of 92 (4%) autoantibody-positive patients with pSS did not carry any risk genotypes compared with 36 of 96 (38%) of controls.

### Genetic associations with Ro/La autoantibody subsets in primary Sjögren syndrome

Of 115 patients with pSS, 19 (16%) were negative and 97 (84%) positive for anti-Ro/La autoantibodies. Seropositive Ro+La patients by enzyme-linked immunosorbent assay were further subdivided into non-precipitating La, ie, Ro+La (ppt–), or precipitating, ie, Ro+La (ppt+), on the basis of a precipitin line formed by anti-La antibodies on CIEP. Therefore, in addition to a seronegative subset, seropositive patients with pSS were classified into one of three serological subsets: anti-Ro alone (19 of 115 = 16%), anti-Ro+La(ppt−) (22 of 115 = 19%) and anti-Ro+La(ppt+) (55 of 115 = 46%). These subgroups are characterised by increasing titre and a more polyclonal autoantibody response in addition to higher rheumatoid factor and IgG levels.^4^ ^32^

**Figure 3:**
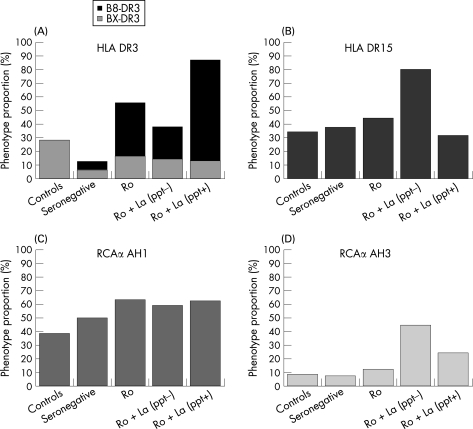
Phenotypic prevalence of HLA DR3 (A), HLA-DR15 (B), RCAα AH1 (C) and RCAα AH3 (D) haplotypes by diversification of the Ro/La autoantibody response within primary Sjögren syndrome (pSS). There were 115 patients with pSS in the study: 19 were seronegative, 19 with anti-Ro only, 22 with anti-Ro+La (ppt−) and 55 with anti-Ro+La (ppt+). The increased prevalence of HLA DR3 in autoantibody-positive pSS is largely B8-DR3 (the prevalence of B8-DR3 in the controls is not known) and there are genetic differences between the pSS autoantibody subgroups.

We, and others^4^ ^5^ have previously demonstrated that HLA-DR3 and DR15 frequencies differ between autoantibody subsets, and this question is also of interest in relation to the RCAα haplotypes. The phenotypic prevalences of DR3, DR15 and RCAα AH1 and AH3 in controls, seronegative pSS and the three pSS autoantibody subsets are displayed in fig 3. While the data are difficult to interpret definitively because of the relatively small sample sizes in three of the serological subsets, some inferences may be drawn.

The increased prevalence of HLA DR3 in autoantibody-positive pSS is largely B8-DR3, which is carried on the 8.1 ancestral haplotype. B8-DR3 is most strongly associated with the polyclonal Ro+La (ppt+) autoantibody response, whereas, as previously reported,^4^ DR15 is almost exclusively associated with the restricted Ro+La (ppt−) response. The RCAα AH1 prevalence, although elevated, is relatively constant in autoantibody-positive patients. The basis for the epistatic interaction between RCAα AH1 and DR3 is most likely restricted to the 8.1 ancestral haplotype, rather than other DR3 containing haplotypes, and may exert a primary influence on the autoantibody response in pSS.  Interestingly, 8.1 ancestral haplotype only one, rather than two or more C4 genes and is therefore associated with a relative C4 deficiency.^34^ However, there are clearly other genes on the MHC 8.1 haplotype that contribute additionally to regulation of the autoantibody response. The RCAα AH3 haplotype has the highest prevalence in patients with anti-La and may therefore potentiate diversification of the autoantibody response.

The genes for C2 are also in the extended MHC region and type 1 C2 deficiency is encoded within the 18.1 haplotype, which carries B18-DR15. There was no evidence of any epistatic interaction between RCAα block haplotypes and DR15. Further, only four B18-DR15 (from a total of 52 DR15) haplotypes were observed in patients with pSS and, as expected, there were no associations.

## DISCUSSION

The GMT haplotyping approach has been used in this study to identify AH of the RCAα block and their associations with pSS, a systemic autoimmune disease. Duplication and copy number variation reveal more genetic diversity than single nucleotide polymorphisms,^19^ ^35^ yet for most haplotyping assays, interpreting the results and defining haplotypes in these regions is difficult. The GMT assay is useful in interrogating complex genomic regions, such as the MHC and RCA blocks, as it requires and utilises these features to efficiently define the genomic polymorphism and AH within the region. The GMT RCAα block haplotyping has revealed extensive haplotypic polymorphism in this region (which also includes CR1-L, MCP and MCP-L genes), with more than 20 AH defined,^25^ although the majority are rare.

In this study we report that relatively frequent RCAα block haplotypes, AH1 and AH3, are associated with pSS, an autoimmune disease with a high prevalence of antinuclear Ro/La autoantibodies, and which shares both clinical and genetic susceptibility overlap with SLE.

The RCAα block contains CR1, CR1-L, MCP and MCP-L genes, and possibly also CR2, although the boundaries of the block have not been precisely defined. Erythrocyte CR1 in humans provides an important mechanism for the non-inflammatory clearance of immune complexes opsonised with C3a and C4b, and genetic variation in CR1 expression and/or function may influence both the predisposition to autoantibody-mediated disease (the clearance hypotheses)^36^ and self-perpetuating autoimmune reactivity resulting from prolonged, immune complex-mediated, type I interferon production.^8^ Although an acquired loss of erythrocyte CR1 expression has been observed in diseases such as SLE and pSS, the phenotypic high/low CR1 expression polymorphism is not associated with SLE,^11^ although the genetic basis for this is not well defined. CR1 expression is downregulated by immune complexes and upregulated by interferon γ in SLE,^37^ and in the mouse, CR1/CR2 expression (encoded by the same gene) is upregulated by BAFF.^38^ Therefore, genetic differences in the compound regulation of CR1 expression are likely to be strongly influenced by the complex inflammatory disease milieu.

In addition to clearance of immune complexes, CR1 and CR2 play a direct, instructive role in setting the threshold for B cell responses to antigen^39^ and potentially, the loss of B cell tolerance,^40^ a fundamental step in the pathogenesis of autoimmune disease. Further, cross-linking of MCP (CD46) with the TCR on naïve CD4+ T cells induces regulatory T cells,^41^ which are critical in the balance between autoimmunity and tolerance.^42^

Finally, complement control proteins may function as receptors for ligands other than complement components. The CR2 receptor is of particular interest in this regard, and a recent study has demonstrated an association between a common CR2 single nucleotide polymorphism based haplotype and SLE.^43^. CR2 is not only a functional receptor for interferon α,^44^ but is also the Epstein–Barr virus (EBV) receptor. There has long been a strongly suspected link between EBV infection and systemic autoimmunity. Intriguingly, molecular mimicry between the EBNA-1 epitope of the EBV virus and the Ro autoantigen has been demonstrated in initiation of the anti-Ro response in patients who ultimately developed SLE.^45^

Similar to HLA haplotypes, RCAα block haplotypes exert an influence on Ro/La autoantibody responses in patients with pSS. Importantly, there was an epistatic interaction between RCAα AH1 and HLA B8-DR3 in patients with pSS with Ro/La autoantibodies, which is indicative of a biological relationship between gene products of these haplotypes and disease pathways. The most likely basis for this epistasis is an interaction between the CR1 receptor and C4, one of its ligands. The genes for C4 are in the extended MHC region. HLA B8-DR3 and a relative C4 insufficiency (C4A*Q0,C4B*1)^34^ are both part of the 8.1 ancestral haplotype. The genetic structure of the C4 region is itself complex and highly polymorphic with both allelic and copy number variation of C4A and C4B genes.^46^ Genetic variation in C4 is known to contribute to disease susceptibility associated with MHC haplotypes^47^^–^49, but other MHC genes are also likely to be involved.^34^ ^48^ In addition to systemic diseases such as pSS and SLE, the MHC 8.1 haplotype is associated with a number of organ-specific autoimmune diseases such as type 1 diabetes mellitus, Hashimoto’s thyroiditis, Graves’ disease, myasthenia gravis and multiple sclerosis. Evaluation of RCAα/MHC interactions in these diseases, and their association with susceptibility, autoantibody production and clinical phenotype would clearly be of great interest.

In addition to demonstrating the utility of the GMT approach for interrogating polymorphism in complex genomic regions, this study has also demonstrated that normal population variation in the RCAα block contributes substantially to susceptibility to systemic autoimmune disease. This is therefore direct evidence of the importance of regulation of complement activation in disease pathogenesis. The differing functional effects of the RCAα block haplotypes have not yet been defined and will be the focus of future research. These are likely to be pleiotropic and may well involve further epistatic genetic interactions. Although the task of unravelling these effects will be highly complex, we predict they will provide important insights into underlying autoimmune disease mechanisms.
